# Detection of plasma EV-associated TRAIL by nanoscale flow cytometry for liver metastasis prediction in PDAC

**DOI:** 10.1007/s44307-026-00102-1

**Published:** 2026-03-04

**Authors:** Chun-Xiang Huang, Jia-Hong Jian, Jun-Sheng Hao, Zi-Wen Zhou, Zhuo-Qun Li, Dong-Ming Kuang, Cai-Yuan Wu

**Affiliations:** Guangdong Province Key Laboratory of Pharmaceutical Functional Genes, MOE Key Laboratory of Gene Function and Regulation, School of Life Sciences, Sun Yat-Sen University, Guangzhou, 510275 China

**Keywords:** Nanoscale flow cytometry, Extracellular vesicles, Pancreatic ductal adenocarcinoma, Liver metastasis

## Abstract

Extracellular vesicles (EVs) mediate tumor–host communication and represent a promising liquid biopsy source for metastasis risk assessment, yet quantitative detection of low-abundance, epitope-defined EV subpopulations in plasma remains technically challenging. Here, we establish a nanoscale flow cytometry workflow on the CytoFLEX platform for sensitive single-EV phenotyping by optimizing violet side scatter (VSSC) triggering, defining an acquisition window that minimizes coincidence or “swarm” effects, and applying fluorescence-based analysis with stringent background controls. Using this framework, we quantified EV-associated tumor necrosis factor-related apoptosis-inducing ligand (TRAIL) at the single particle level, with good inter-assay reproducibility (CV ~ 11–13%), and resolved low-abundance TRAIL⁺ EVs at approximately 1% abundance within total EV events. Due to the low abundance of EV-associated TRAIL in pancreatic ductal adenocarcinoma (PDAC) plasma, ELISA lacked sufficient analytical sensitivity to accurately reflect EV-associated TRAIL levels, whereas flow-based enumeration preserved quantitative resolution. Clinically, plasma EV-associated TRAIL was significantly elevated in PDAC patients with liver metastasis and demonstrated predictive utility for postoperative liver metastatic recurrence (AUC = 0.766). These results support nanoscale flow cytometry as a robust platform for plasma EV biomarker profiling and identify EV-associated TRAIL as an informative indicator of liver metastatic risk in PDAC.

**Supplementary Information:**

The online version contains supplementary material available at 10.1007/s44307-026-00102-1.

## Introduction

Extracellular vesicles (EVs) are nanoscale, membrane-bound vesicles secreted by most cell types and carry diverse functional cargo, including proteins, lipids, and nucleic acids (Jeppesen et al. 2019). Tumor-derived EVs have emerged as key mediators of intercellular communication that can remodel distant tissues and reshape the systemic microenvironment to facilitate metastatic progression (Liu et al. 2016; Spinelli et al. 2024). Accordingly, resolving and quantifying defined tumor-associated EV subpopulations in circulation is of substantial interest for minimally invasive liquid biopsy applications, including early detection and prediction of distant metastasis (Cohen et al. 2018).

Among EV-associated factors, tumor necrosis factor–related apoptosis-inducing ligand (TRAIL) has been linked to metastatic progression (Wu et al. 2025). In our previous study, we found that EV-associated TRAIL promotes pre-metastatic niche formation, and an ELISA-based measurement of plasma EV-associated TRAIL could predict postoperative lung metastasis in hepatocellular carcinoma (HCC) (Wu et al. 2025). A key limitation of ELISA, however, is that quantification relies on interpolating optical density values from a standard curve, which restricts analytical sensitivity. When TRAIL levels fall near the lower end of the assay’s usable range, the readout becomes compressed and less reliable, making it difficult to resolve modest but potentially meaningful differences. These considerations motivated us to develop a more sensitive method that can directly quantify TRAIL-positive EV subpopulations in plasma.

Pancreatic ductal adenocarcinoma (PDAC) is an aggressive malignancy with a high propensity for early dissemination (Rozengurt and Eibl 2026), and the liver represents the predominant site of distant metastasis (Stoop et al. 2025; Cohen et al. 2018). Accurate prediction of liver metastasis risk would have important implications for clinical decision-making and postoperative surveillance. However, the tumor microenvironment in PDAC is characterized by abundant fibroblasts and extensive extracellular matrix deposition, together with a low proportion of tumor cells, which may reduce the fraction of tumor-derived EVs within the circulating EV pool, creating a major analytical barrier for detecting low-abundance EV-associated biomarkers.

Flow cytometry offers a potential route toward single-EV phenotyping and subpopulation-resolved quantification (Conroy et al. 2023; Welsh et al. 2023), yet EV detection in plasma is challenged by the small size of EVs, the presence of abundant non-EV nanoparticles (e.g., lipoproteins and protein complexes) with overlapping biophysical properties, and concentration-dependent coincidence (“swarm” effects) that distort event rates and fluorescence/scatter signals (Liu et al. 2022; Libregts et al. 2018). Here, we establish and validate a nanoscale flow cytometry workflow on the CytoFLEX platform by optimizing scatter triggering, implementing rigorous controls to mitigate swarm and background interference, and integrating fluorescence-based phenotyping to quantify defined EV subsets. Applying this workflow to PDAC patient cohorts, we demonstrate sensitive quantification of plasma EV-associated TRAIL and show that EV-associated TRAIL provides strong predictive performance for liver metastasis, supporting its potential utility for metastasis risk stratification.

## Methods

### Patients and specimens

A total of 165 peripheral blood samples from PDAC patients and 20 peripheral blood samples from HCC patients were collected for this study. Patients with concurrent autoimmune disease, HIV, or syphilis were excluded. 47 PDAC patients without liver metastasis, 33 PDAC patients with liver metastasis (cohort 1; Table S1), and 20 HCC patients were obtained from the Third Affiliated Hospital of Sun Yat-sen University between March 2021 and January 2025. These samples were used to assess the association between plasma EV-associated TRAIL levels and metastasis, and were also used for ROC analysis. Another 85 patients who underwent surgical resection at Sun Yat-sen University Cancer Center between August 2016 and August 2019 with complete follow-up data (cohort 2; Table S2) were enrolled for validation of the prognostic potential of EV-associated TRAIL in predicting liver metastasis. None of the patients showed visible metastasis at the time of surgery, and none had received anticancer therapy prior to sampling. 20 control peripheral blood samples were obtained from healthy volunteers registered in the physical examination center of the Third Affiliated Hospital of Sun Yat-sen University. All samples were anonymously coded in accordance with local ethical guidelines (as stipulated by the Declaration of Helsinki). Written informed consent was obtained from the patients, and the protocol was approved by the Ethical Review Board of Sun Yat-sen University.

### Cell culture and generation of TRAIL-overexpressing HEK293T cells

HEK293T cells were obtained from the Cell Bank of the Type Culture Collection of the Chinese Academy of Sciences (Shanghai, China) and used within 6 months after purchase. Cells were routinely tested for mycoplasma contamination by single-step PCR and cultured in Dulbecco’s modified Eagle’s medium (DMEM; Gibco) supplemented with 10% fetal bovine serum (FBS; Gibco) at 37 °C in a humidified incubator with 5% CO₂.

To generate TRAIL-overexpressing HEK293T cells, the human TRAIL coding sequence was amplified by PCR from cDNA, verified by Sanger sequencing, and cloned into the pCDH-CMV-MCS vector (System Biosciences). For transient transfection, HEK293T cells in logarithmic growth phase were seeded in 10-cm dishes at 30–50% confluency. Plasmid DNA (7 μg) was diluted in 1 mL serum-free DMEM, mixed gently, and combined with 21 μL polyethylenimine (PEI). The mixture was vortexed briefly and incubated at room temperature for 15 min to allow DNA–PEI complex formation. Cells were washed by replacing the medium with 9 mL serum-free DMEM, and the DNA–PEI complexes were added dropwise with gentle swirling. After 4 h incubation at 37 °C with 5% CO₂, the transfection medium was replaced with 10 mL pre-warmed complete DMEM containing 10% FBS, and cells were maintained under standard culture conditions.

### EVs isolation from cell cultures and plasma

When the cells reached 60–80% confluence, they were transferred to normal DMEM without FBS for 12 h. The supernatants were harvested for EVs isolation. The medium was then collected and centrifuged at 300 g for 10 min, followed by a centrifugation step of 2,000 g for 10 min to remove cellular debris. Subsequently, the supernatant was filtered using a 0.22 μm filter. The collected media was then ultracentrifuged at 100,000 g for 2 h at 4 °C, and the supernatant was discarded.

 EVs from plasma were isolated as described by Clotilde (Théry et al. 2006). To obtain plasma for EV isolation, human blood was centrifuged at 500 g for 5 min, 2,000 g for 15 min and 10,000 g for 20 min at 4 °C to remove cells, debris and large vesicles. 1 mL plasma were diluted with a 50-fold volume of PBS and centrifuged at 2,000 g for 30 min, followed by a second centrifugation at 12,000 g for 45 min. The supernatant was then filtered using a 0.22 μm filter. Finally, the supernatant was ultracentrifuged at 110,000 g for 2 h at 4 °C, and the supernatant was discarded.

EV preparations were characterized by NTA and TEM, and were further validated by immunoblotting for TSG101 (a cytosolic/ESCRT-associated EV marker) prior to downstream phenotyping. CD63 (a canonical EV membrane marker) was evaluated by nanoscale flow cytometry for single-EV phenotyping.

### Flow cytometric analysis of 293T cells and nanoscale particles

Wild-type or TRAIL-overexpressing HEK293T cells were stained with PE-labeled anti-TRAIL antibody (12–9927-42, eBioscience) according to the manufacturer’s instructions and analyzed on a CytoFLEX LX flow cytometer (Beckman Coulter).

For nanoscale particle analysis, 100, 200, and 300 nm fluorescent polystyrene beads (FluoSpheres, Thermo Fisher Scientific) were used as size/sensitivity references and for daily performance verification prior to sample acquisition. All buffers used for nanoscale flow cytometry (PBS and staining/dilution buffers) were filtered through a 0.02 μm filter (6809–2002, Cytiva) to minimize background particles. EVs derived from HEK293T cells or isolated from plasma, together with reference beads, were analyzed on the CytoFLEX LX using a dual-scatter configuration. Event triggering was applied to violet side scatter area (VSSC-A; 405-SSC) to maximize sensitivity for nanoscale particles, whereas 488-nm side scatter area (SSC-A) was used as the primary scatter channel for downstream visualization and quantification. Unless otherwise specified, scatter and fluorescence signals were quantified using area (A) parameters.

Instrument gains were set as follows: FSC (gain 99), SSC (gain 153), VSSC (gain 150), PE (gain 1098), and APC (gain 2500). The VSSC-A trigger threshold was set to 30,000, and threshold/gating performance was verified using 100- and 200 nm beads. The threshold was further confirmed by acquiring particle-free PBS, with a target background event rate of ≤ 100 events/s.

Samples were acquired at fast mode (60 μL/min). To minimize coincidence (“swarm”) effects, sample concentration was optimized by serial dilution of 100 nm fluorescent beads while monitoring event rate together with SSC-A and fluorescence intensity; EV samples were diluted as needed to maintain acquisition within the established linear range (~ 3,000–6,000 events/s). During acquisition, samples were loaded directly without vortex or additional mixing.

For fluorescence-based phenotyping of nanoscale particles, positive gates were defined using matched isotype controls processed in parallel and acquired under identical instrument settings, in addition to particle-free PBS controls. To reduce carryover, the system was flushed with 0.02 μm filtered PBS for 2–5 min before and after each run (and between samples when necessary). Each sample was acquired at a sample flow rate of 60 μL/min.

### Nanoparticle tracking analysis

Concentration and size distribution of isolated exosomes were assessed by nanoparticle tracking analysis (NTA) using NanoSight NS300 instrumentation (NanoSight, Amesbury, UK). Exosome samples were diluted with PBS at a range of concentrations between 4 × 10^8^ and 8 × 10^8^ particles per milliliter in a total volume of 1 mL. Each sample was continuously run through a flow-cell top-plate set up to 23.3 °C using a syringe pump at a rate of 25 μL/min. At least three videos of 30 s documenting Brownian motion of nanoparticles were recorded and at least 1000 completed tracks were analyzed by NanoSight software (NTA 2.3.5).

### Electron microscopy

Isolated EVs were fixed in 2% paraformaldehyde in 0.1 M phosphate buffer overnight at 4 °C. The samples were then placed on formvar-carbon-coated grids and air dried for 20 min. After being rinsed with PBS, grids were transferred to 1% glutaraldehyde for 5 min and washed with distilled water. The grids were first contrasted with uranyl-oxalate solution, followed by contrasting and embedding in a mixture of 4% uranyl acetate and 2% methylcellulose (1:9 ratio). The grids were air dried and visualized with a JEM 1400 electron microscope (JEOL USA, Peabody, MA) at 120 kV.

### Bulk EVs staining with DiR

EVs were fluorescently labeled with the near-infrared lipophilic dye DiR (1,1′-dioctadecyl-3,3,3′,3′-tetramethylindotricarbocyanine iodide) (HY-D1048, MCE). Purified EVs were resuspended in particle-free PBS and incubated with DiR at a final concentration of 5 μM for 20–30 min at room temperature in the dark with gentle mixing. To remove free (unincorporated) dye, labeled EVs were washed by ultracentrifugation at 100,000 g for 70 min at 4 °C, resuspended in particle-free PBS, and washed once more under the same conditions. A dye-only control (DiR processed in parallel without EVs) and an unlabeled EV control were prepared and subjected to the same washing procedure.

### CD63 and TRAIL staining

EVs were stained with APC-labeled anti-CD63 antibody (353,008, biolegend) and PE-labeled anti-TRAIL antibody (12–9927-42, eBioscience) for 25 min at 4 °C. Before staining, antibody aggregates were removed by 16,000 g centrifugation for 8 min. Staining was terminated by adding 12 mL PBS and then filtered using a 0.22 μm filter, ultracentrifuged at 110,000 g for 2 h at 4 °C, and the supernatant was discarded.

### Immunoblotting

Proteins were extracted as described previously (Wu et al. 2025). In brief, protein samples were resolved on a 10% SDS-PAGE gel under reducing conditions, transferred to polyvinylidene difluoride membranes and blocked with 3% (w/v) Bovine serum albumin (BSA, Sigma-Aldrich). Blots were incubated with antibodies (anti-CD63, ab134045, abcam; anti-TSG101, ab83, abcam; anti-TRAIL, ab231265, abcam) followed by HRP-conjugated anti-IgG antibodies (Cell Signaling Technology) and detected with ECL kit (Thermo Fisher Scientific) by Chemiluminescence Imaging System (Syngene, GeneGnome XRQ).

### Enzyme-linked immunosorbent assay (ELISA) for EVs TRAIL detection

For detection of TRAIL on human plasma EVs, EVs from 1 mL plasma were coated with 50 μL coating buffer (50 mM NaHCO_3_, pH 9.6) per well overnight at 4 °C. Free binding sites were blocked using Carbo-Free Blocking Solution (Vector Laboratories) for 1 h. Then 50 μL of antibody against TRAIL (ab9959, abcam) were added and incubated for 4 h at room temperature, followed by incubation with 50 μL HRP conjugated anti-IgG antibodies (Cell Signaling Technology) for 1 h at room temperature. Thereafter, wells were developed with 3,3’,5,5’-Tetramethylbenzidine substrate (eBioscience) for 15 min and stopped with 25 μL 1 M H_2_SO_4_. Wells were read at 450 nm minus 570 nm with a microplate reader (Molecular Devices, SpectraMax i3x). Wells between each incubation were washed with Tris Buffered Saline with 0.1% Tween-20. Recombinant human TRAIL protein (375-TL, R&D Systems) was used to make a standard curve. The result of standard curve demonstrated that the established ELISA exhibited a reliable linear detection ranging from 0.8 to 100 ng/mL.

### Logistic regression model and ROC analysis

We developed a logistic regression model to predict binary outcomes based on the percentage of TRAIL⁺ EVs among total plasma EVs. The percentage of TRAIL⁺ EVs was mean-centered and scaled (z-transformed) using the parameters estimated from the full dataset. Model discrimination was evaluated by receiver operating characteristic (ROC) analysis, and the area under the ROC curve (AUROC) was calculated. Sensitivity, specificity, accuracy, positive predictive value (PPV), and negative predictive value (NPV) were derived from the confusion matrix. The optimal classification cutoff was determined using Youden’s J statistic.

### Quantification and statistical analysis

Results are presented as mean ± SEM. All statistical tests were two-sided. Data normality was assessed using the Shapiro–Wilk test. For comparisons between two groups, Student’s t test was used. For comparisons among three or more groups, one-way analysis of variance (ANOVA) was performed, followed by an appropriate post hoc multiple-comparison test (e.g., Tukey’s test for all pairwise comparisons or Dunnett’s test for comparisons against a single control group), as indicated in the figure legends. A *P* value < 0.05 was considered statistically significant.

## Result

### Establishment of a nanoscale EVs workflow on CytoFLEX

**Fig. 1:**
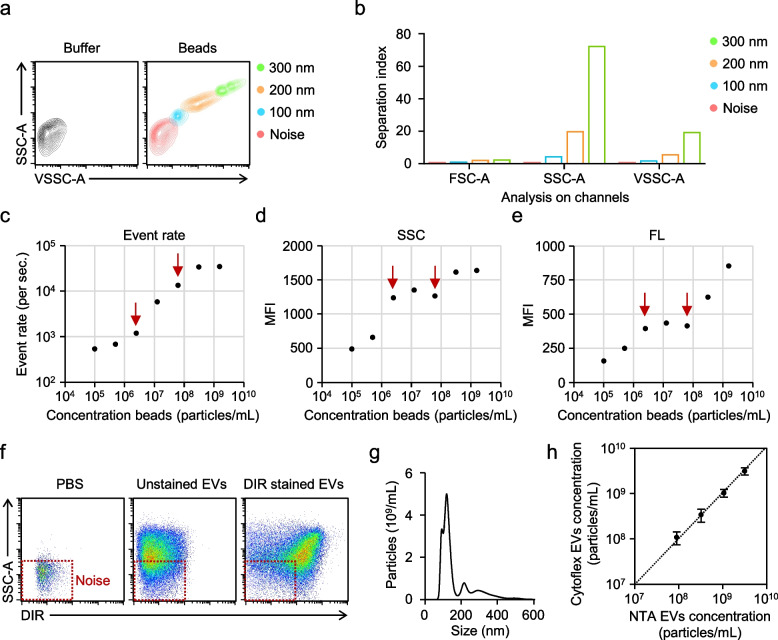
Detection and quantitative analysis of EVs by flow cytometry. **a**, Reference noise, 100, 200, and 300 nm polystyrene beads analysis by CytoFLEX. **b**, Separation index of noise, 100, 200, and 300 nm beads in 488-FSC, 488-SSC and 405-VSSC channel. **c-e**, Serial dilutions from a highly concentrated 100 nm bead suspension were prepared and analyzed using a sample flow rate of 60 μL/min. Analysis of event rate (**c**), side scatter (SSC) (**d**) and fluorescence (FL) (**e**). **f**, Representative plot of PBS, unstained HEK293T EVs, and DiR-stained HEK293T EVs analyzed by CytoFLEX. **g**, Representative plot of HEK293T EV diameter distribution and concentration obtained using nanoparticle tracking analysis (NTA). **h**, Comparison of HEK293T EV enumeration using NTA and CytoFLEX. Representative plots are shown (**f** and **g**); experiments were repeated independently three times with similar results (*n* = 3). Data represent mean ± SEM of three (**h**) independent experiments, statistical analysis was performed using Student’s t test (**h**)

Logically, the optimal scatter channel for threshold triggering should enable reliable detection of nanoscale particles above background noise. To determine the most suitable triggering parameter on the CytoFLEX, nanobeads were acquired using different scatter channels, and signal separation from reference noise was evaluated across detectors. Consistent with the higher sensitivity of short-wavelength side scatter for small particles, the Violet SSC (VSSC) channel enabled detection of the smallest bead populations and was therefore selected for threshold triggering (Fig. 1a). When signal separation was quantitatively assessed across analysis channels, however, 488-nm SSC provided superior discrimination between bead populations and reference noise, as reflected by higher separation index values compared with FSC or VSSC (Fig. 1b). Accordingly, VSSC was used for event triggering to ensure efficient detection of nanoscale particles, whereas 488-SSC was used as the primary analysis channel to achieve optimal resolution and dynamic range. Together, these data support a dual-channel configuration on the CytoFLEX—VSSC for sensitive triggering and 488-SSC for downstream analysis—for robust detection and quantitative evaluation of small extracellular vesicles.

Nanoscale flow cytometry is particularly susceptible to coincident (“swarm”) detection at high particle concentrations, whereby multiple particles are recorded as a single event, leading to an underestimation of event rates. To define a working range that minimizes swarm, serial dilutions of 100-nm fluorescent beads were acquired at 60 μL/min while event rate, SSC, and fluorescence (FL) intensity were monitored in parallel. A linear relationship between particle concentration and event rate was observed only within the ~ 10⁷ particles/mL range, corresponding to approximately 3,000–6,000 events/s, where dilution produced the expected proportional decrease in event rate (Fig. 1c). At higher concentrations outside this linear range, event rates deviated from proportionality and both SSC and fluorescence intensity progressively increased (Fig. 1d, e), consistent with signal summation from coincident particles. Accordingly, subsequent EV measurements were performed within this linear concentration and event-rate window to minimize coincidence-related bias and ensure quantitative reliability.

To validate the applicability of the established CytoFLEX nano-flow workflow to biological nanoparticles, fluorescently labeled EVs derived from HEK293T cells were analyzed using the optimized triggering and acquisition settings described above. The labeled EV population was clearly resolved from the system reference noise, with the distinction most apparent in two-dimensional scatter–fluorescence analysis (Fig. 1f). Detergent lysis substantially reduced EV-associated fluorescence signals, supporting that the detected events were membrane-associated vesicles (Supplementary Fig. 1). In parallel, NTA measurements of EV size distribution and particle concentration were consistent with the flow-based event characteristics and showed good concordance across platforms (Fig. 1g, h), further supporting the feasibility and robustness of this workflow for nanoscale EV detection and quantitative analysis.

### Detection of CD63 and TRAIL on extracellular vesicles using flow cytometry

While detection of non-specific EV labels is achievable on many platforms to varying extents, robust detection of defined surface epitopes with conventional antibodies remains a central goal of EV flow cytometry. Single-EV phenotyping provides a powerful approach to interrogate EV heterogeneity in both cell-derived preparations and patient plasma. Using the optimized CytoFLEX nano-flow workflow, we evaluated the detection of CD63 and membrane-associated TRAIL on EVs derived from TRAIL-overexpressing HEK293T cells. CD63 is a canonical EV membrane marker. TRAIL was previously identified in our work as a functional mediator of pro-metastatic niche formation in distant organs and a potential biomarker for metastasis risk stratification.

**Fig. 2:**
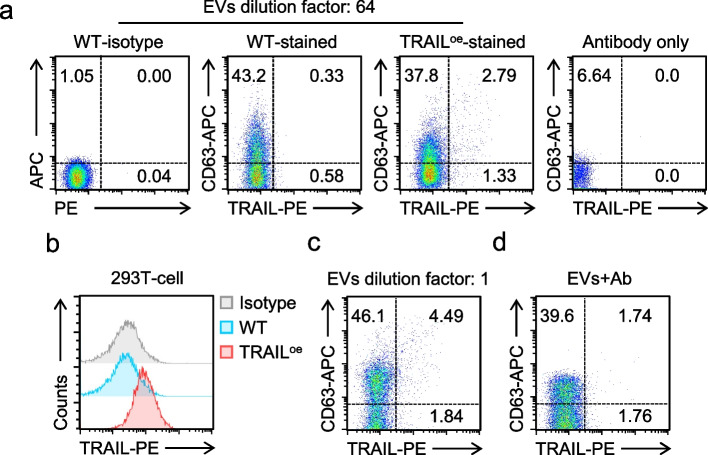
Detection of CD63 and TRAIL on EVs by flow cytometry. **a**, EVs from wild-type (WT) or *TRAIL*-overexpressing (TRAIL^oe^) HEK293T cells were stained with PE-labeled anti-TRAIL antibody and APC-labeled anti-CD63 antibody. The 64-fold diluted samples were analyzed by flow cytometry. An antibody-only control (antibodies processed in parallel without EVs) was included to assess background signals from free antibodies. **b**, Flow cytometric analysis of TRAIL in WT or TRAIL^oe^ HEK293T cells. **c**, Flow cytometric analysis of TRAIL and CD63 in undiluted EVs from TRAIL^oe^ HEK293T cells, illustrating increased coincidence/“swarm” effects compared with the optimized dilution condition in (**a**). **d**, Analysis of EV samples without post-staining washing to remove excess staining antibodies, showing elevated fluorescence background compared with the washed condition in (**a**). Representative plots are shown (**a**-**d**); experiments were repeated independently three times with similar results (*n* = 3)

EVs from both wild-type and TRAIL-overexpressing (TRAIL^oe^) HEK293T cells were co-stained with anti-TRAIL-PE and anti-CD63-APC. Matched isotype controls were used to define the negative population and to set the fluorescence positivity gates. In addition, an antibody-only control showed minimal background events, supporting that the detected fluorescence signals were not driven by free antibodies (Fig. 2a). Under these conditions, a distinct CD63-positive population was observed (Fig. 2a); however, TRAIL-positive events were predominantly observed in TRAIL^oe^ preparations, whereas signals in wild-type EVs were near background (Fig. 2a). These staining profiles were consistent across independent experiments, demonstrating high inter-assay reproducibility (Table S3). This observation is consistent with the minimal expression of membrane-associated TRAIL in wild-type HEK293T cells and supports the specificity of the detected signals (Fig. 2b). Importantly, when highly concentrated EV suspensions were acquired, coincidence (“swarm”) artifacts produced an apparent increase in CD63-positive and TRAIL-positive events (Fig. 2c). Accordingly, reliable identification and quantification of antibody-defined EV subsets required measurements within a dilution window in which fluorescence signals remained stable. In addition, omission of a post-staining wash step resulted in a marked rightward shift of the reference-noise fluorescence distribution (Fig. 2d), thereby masking true CD63-positive and TRAIL-positive events and reducing detection sensitivity. Removal of free antibodies reduced background and improved the resolution of TRAIL-positive EVs in HEK293T-derived samples.

### PDAC plasma EVs preparation and flow analysis

**Fig. 3:**
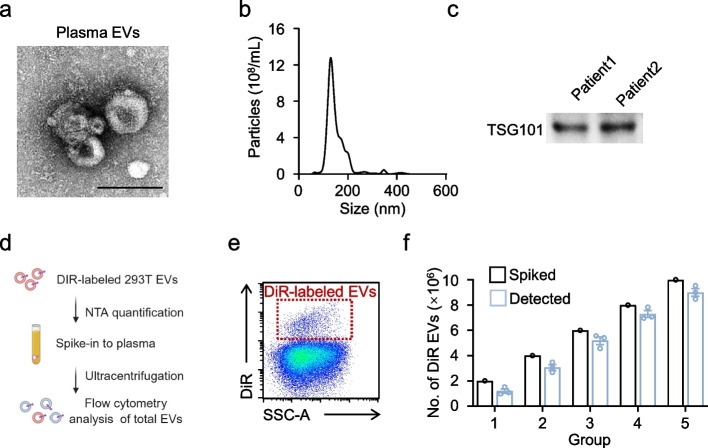
Characterization and flow cytometric analysis of plasma EVs from PDAC patients. **a**, Representative image of transmission electron photomicrographs of plasma EVs from PDAC patients. Scale bar, 200 nm. **b**, NTA of plasma EVs from HCC patients. **c**, WB analysis for the expression of specific protein markers (TSG101) in plasma EVs from PDAC patients. **d**, **e**, DiR-labeled HEK293T-derived EVs were quantified by NTA and then spiked into unstained plasma at defined inputs. Spiked and endogenous particles were subsequently co-isolated by ultracentrifugation as described in (**d**). DiR-labeled EVs were analyzed by flow cytometry (**e**). **f**, The comparison of spiked-in DiR-labeled EVs and DiR-labeled EVs detected by flow cytometry. Representative plots are shown (**a**-**c**,** e**, and** f**); experiments were repeated independently three times with similar results (*n* = 3)

We next evaluated the performance of the CytoFLEX nano-flow workflow for detecting plasma EVs from patients with PDAC. Plasma-derived EVs were isolated and enriched by ultracentrifugation. To confirm successful EV isolation prior to downstream analyses, EV preparations were characterized by morphology, size distribution, particle concentration, and EV-enriched protein markers. Transmission electron microscopy (TEM) revealed typical nanoscale, cup-shaped vesicular structures (Fig. 3a), consistent with previous reports. NTA showed that PDAC plasma EVs exhibited a size distribution comparable to HEK293T-derived EVs, with a mean diameter of 157 ± 80 nm (Fig. 3b) and an approximate concentration of 10^11^ particles per mL of plasma. In addition, immunoblotting confirmed the presence of the EV marker TSG101 (Fig. 3c), supporting the suitability of these preparations for subsequent experiments.

Given that plasma contains abundant lipoproteins (e.g., HDL/LDL/VLDL) that overlap with EVs in size and can confound scatter-based nanoparticle detection, we further examined whether fluorescence-based nano-flow cytometry can reliably quantify a defined EV subpopulation in plasma processed by ultracentrifugation. For this purpose, DiR-labeled HEK293T-derived EVs were quantified by NTA and then spiked into unstained plasma at defined inputs. Spiked and endogenous particles were subsequently co-isolated by ultracentrifugation and analyzed by nano-flow cytometry (Fig. 3d). DiR-positive events were readily detected using the CytoFLEX workflow (Fig. 3e). Although the absolute number of recovered DiR-positive events was modestly lower than the input (Fig. 3f), the measured DiR-positive counts scaled linearly with the spiked EV input across the dilution series (Fig. 3f). Collectively, these data indicate that ultracentrifugation-based enrichment coupled with fluorescence-triggered nano-flow analysis provides a quantitative readout that faithfully captures relative abundance changes of specific EV subsets within the plasma matrix.

### Comparative performance of nanoscale flow cytometry and ELISA for plasma EV-associated TRAIL detection in PDAC

**Fig. 4:**
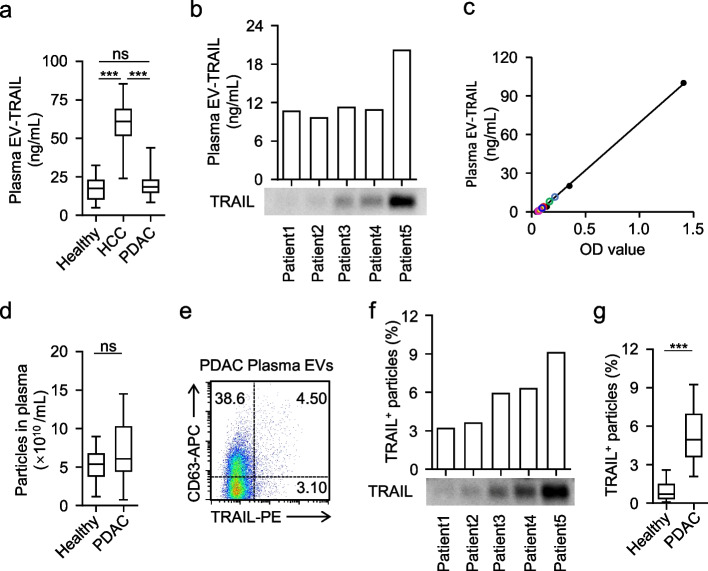
Comparison of plasma EV-TRAIL detection in PDAC patients by flow cytometry and ELISA**. a**, Concentrations of plasma EV-TRAIL in healthy donors, HCC patients, and PDAC patients were detected by ELISA (each *n* = 20). **b**, ELISA analysis (top) and immunoblot (bottom) showing the comparison of plasma EV-TRAIL levels from 5 PDAC patients. **c**, TRAIL ELISA standard curve (black; points and fitted line). Colored points indicate plasma EV samples from individual PDAC patients plotted on the curve for TRAIL quantification. **d**, Concentrations of plasma EVs in healthy donors and PDAC patients were detected by NTA (each *n* = 20). **e**, Plasma EVs from PDAC patients were stained with PE-labeled anti-TRAIL antibody and APC-labeled anti-CD63 antibody and analyzed by flow cytometry. **f**, Flow cytometry analysis (top) and immunoblot (bottom) showing the comparison of plasma EV-TRAIL levels from 5 PDAC patients. **g**, Percentage of TRAIL^+^ EVs in total plasma EVs from healthy donors and PDAC patients (each *n* = 20). Representative plots are shown (**e**); experiments were repeated independently three times with similar results (*n* = 3). Data represent 4 independent experiments (**a**, **d**, and **g**). ***p < 0.001. Statistical analysis was performed using one-way ANOVA with Tukey’s post-test (**a**, **d**, and **g**)

In our previous study, we developed an ELISA-based assay for quantifying EV-associated TRAIL in plasma (Wu et al. 2025). Using this assay, we observed significantly higher plasma EV-associated TRAIL levels in patients with HCC than in healthy controls, and EV-associated TRAIL measurements showed strong predictive performance for postoperative lung metastasis in HCC. We then applied the same ELISA workflow to PDAC plasma EVs. In contrast to HCC, EV-associated TRAIL levels measured by ELISA in PDAC showed no significant elevation relative to healthy controls and were markedly lower than those detected in HCC (Fig. 4a). Notably, ELISA-derived EV-associated TRAIL signals showed limited concordance with immunoblotting (Fig. 4b). Most OD values detected in PDAC plasma samples were clustered near the lower end of the standard curve (Fig. 4c), indicating that measurements were close to the assay’s lower limit of quantification and thus susceptible to reduced accuracy and compression of between-group differences. Consistent with this interpretation, NTA revealed only a modest increase in total plasma EV particle concentrations in PDAC (Fig. 4d), suggesting that tumor-derived EVs may constitute only a small fraction of the circulating EV pool. This is consistent with the PDAC tumor microenvironment, which is characterized by abundant fibroblasts, extensive extracellular matrix deposition, and a low proportion of tumor cells. By contrast, HCC typically exhibits a higher tumor cell content, which may contribute to a greater tumor-derived EV signal in plasma. Together, these observations suggest that the ELISA workflow may have insufficient analytical sensitivity to resolve PDAC-associated differences in plasma EV-associated TRAIL.

We next quantified plasma EV-associated TRAIL using nanoscale flow cytometry. This approach enabled discrimination of CD63 single-positive, TRAIL single-positive, and CD63/TRAIL double-positive EV subsets (Fig. 4e). Notably, compared with HEK293T-derived EVs, PDAC plasma EVs showed a marked increase in the TRAIL single-positive fraction (from ~ 1.3% to ~ 3.1%) (Figs. 2a, 4e), consistent with our recent evidence that, in tumors, TRAIL incorporation into EVs is predominantly ESCRT-dependent rather than CD63-dependent. We then compared inter-patient variation in the proportion of TRAIL-positive EVs. The flow cytometry–based measurements showed trends consistent with immunoblotting results (Fig. 4f). Importantly, EV-associated TRAIL levels were significantly higher in PDAC patients than in healthy controls (Fig. 4g). Collectively, these findings support nanoscale flow cytometry as a more sensitive and representative approach for assessing EV-associated TRAIL in PDAC plasma and provide a basis for subsequent clinical association analyses.

### Predictive performance of plasma EV-associated TRAIL for liver metastasis in PDAC

**Fig. 5:**
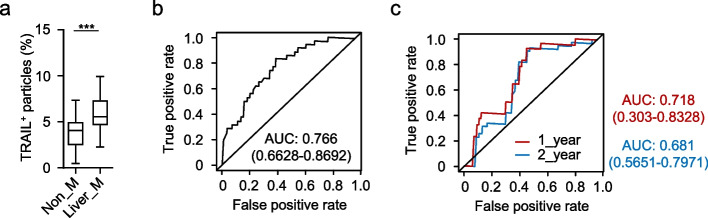
Plasma EV-TRAIL accurately predicts liver metastasis in PDAC patients. **a**, Percentage of TRAIL^+^ EVs in total plasma EVs from PDAC patients without (Non_M) or with (Liver_M) liver metastasis was detected by flow cytometry (Non_M, *n* = 47; Liver_M, *n* = 33). **b**, Receiver operating characteristic (ROC) analysis based on plasma EV-TRAIL for predicting liver metastasis in 80 PDAC patients. **c**, ROC analysis based on plasma EV-TRAIL for predicting liver metastasis within 1 and 2 years post-surgery in 85 PDAC patients

In our previous studies, we demonstrated that EV-associated TRAIL plays a critical role in shaping the pre-metastatic niche (Wu et al. 2025), and the liver represents the predominant site of distant metastasis in PDAC. We therefore quantified plasma EV-associated TRAIL levels in 47 PDAC patients without liver metastasis and 33 patients with liver metastasis (Table S1). Markedly elevated levels of plasma EV-associated TRAIL were observed in patients with liver metastasis compared with those without metastatic disease (Fig. 5a). Using this cohort, we constructed a predictive model for liver metastasis in PDAC. Receiver operating characteristic (ROC) analysis showed that circulating EV-associated TRAIL effectively discriminated metastatic from non-metastatic patients, yielding an area under the curve (AUC) of 0.766 (Fig. 5b).

**Fig. 6:**
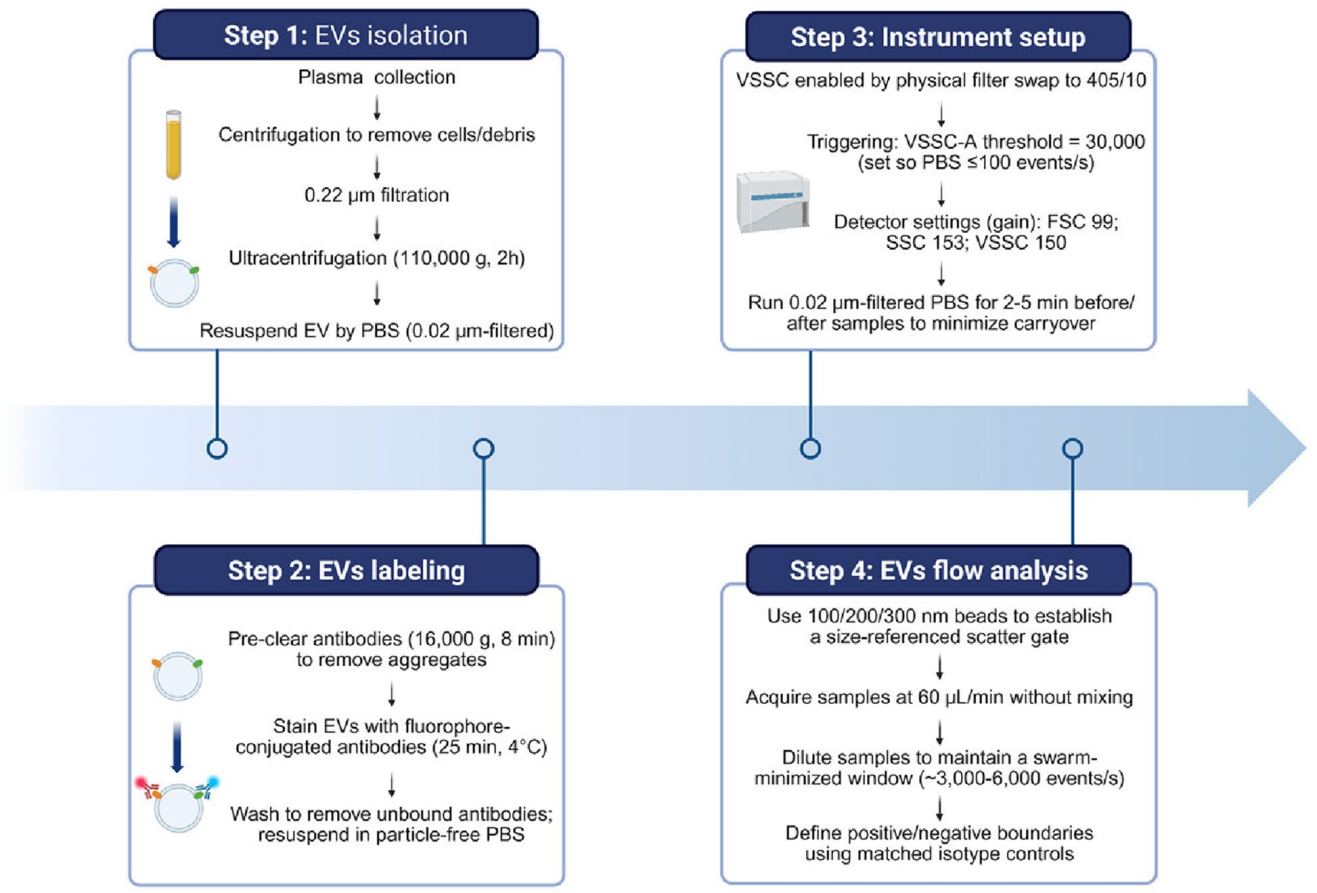
Schematic overview of the CytoFLEX-based nanoscale flow cytometry workflow for single-EV phenotyping. EVs were isolated from plasma by ultracentrifugation and resuspended in particle-free buffer, labeled with fluorophore-conjugated antibodies with removal of antibody aggregates and post-staining washing, and then analyzed on the CytoFLEX LX using VSSC triggering under a defined acquisition window to minimize coincidence (“swarm”) effect. Instrument performance and gating were standardized using 100/200/300 nm fluorescent beads, and positive/negative boundaries were defined using matched isotype controls

To further assess the prognostic value of EV-associated TRAIL, we evaluated the model in an independent cohort of 85 PDAC patients who had no radiologically detectable metastasis at the time of surgical resection (Table S2). Plasma samples were collected on the day of surgery. Time-dependent ROC analysis showed that circulating EV-associated TRAIL provided the strong predictive performance within the first year after surgery, with AUC values of 0.718 at 1 year and 0.681 at 2 years (Fig. 5c). Collectively, these results support the utility of flow cytometry-based plasma EV-associated TRAIL quantification (Fig. 6) as an early warning biomarker for liver metastasis in PDAC patients.

## Discussion

The reliable detection and quantification of low-abundance, tumor-associated EV subpopulations in plasma remain a central analytical challenge for the development of EV-based liquid biopsy strategies (Salmond et al. 2021). Although recent advances in high-sensitivity flow cytometry, microfluidic capture systems, and affinity-based enrichment methods have expanded the methodological landscape for EV analysis (Pham et al. 2024), robust identification of epitope-defined EV subsets in complex biological fluids is still constrained by background interference, particle coincidence, and limited analytical sensitivity (Conroy et al. 2023; Lin et al. 2025; Welsh et al. 2020a, b).

Many published nanoscale flow cytometry workflows have been developed on instruments specifically configured for small particle detection. Although these platforms can offer high sensitivity, they often require stringent, instrument-dependent calibration procedures. By contrast, the CytoFLEX platform is widely used for routine cellular flow cytometry, yet standardized and reproducible strategies for accurate EV detection and phenotyping on CytoFLEX remain less well established. In this study, we developed a standardized nanoscale flow cytometry workflow on the CytoFLEX platform. Our results highlight that the triggering strategy and acquisition conditions are critical determinants of nanoscale analytical performance (Welsh et al. 2020a, b; Bettin et al. 2023; McVey et al. 2018). We also show that at elevated particle concentrations, coincidence and “swarm” effects can contribute to false-positive signals. By defining an acquisition window in which event rates and fluorescence intensities scale linearly, our workflow translates recommendations for EV flow cytometry (Liu et al. 2022; Arraud et al. 2016; Nolan 2015) into practical, quantitative operating criteria. Importantly, because this workflow is implemented on the widely available CytoFLEX platform and reports acquisition settings and quality control criteria explicitly, it should facilitate reproducible adoption across laboratories.

Many methodological studies demonstrate nanoscale performance primarily using reference beads or high-abundance/overexpression models; however, clinical plasma samples such as PDAC represent a substantially more challenging low-abundance setting. Under these conditions, ELISA-based quantification of EV-associated TRAIL was constrained by measurements clustering near the lower limit of quantification and showed limited concordance with EV-associated TRAIL abundance estimated by immunoblotting. In contrast, direct enumeration of TRAIL-positive EVs by nanoscale flow cytometry preserved quantitative resolution at low analyte abundance, thereby enabling discrimination of clinically relevant differences and supporting association with liver metastatic risk in PDAC. This result indicates that, in tumor types with low levels of circulating tumor-derived EVs, bulk EV measurements may have limited sensitivity and can obscure clinically relevant differences. These data support the use of single-particle, subpopulation-based analysis and are in line with previous studies reporting variable performance of bulk EV assays in low-abundance clinical samples. At the biological level, the observation that PDAC plasma EVs showed a marked increase in the TRAIL single-positive fraction compared with HEK293T-derived EVs is consistent with accumulating evidence that EVs originate from multiple, partially distinct biogenetic pathways, including ESCRT-dependent routes that do not obligatorily intersect with tetraspanin-enriched compartments (Buntsma et al. 2023; Colombo et al. 2014; Mathieu et al. 2019).

Although recent studies have advanced the development of circulating EV-based biomarkers in PDAC (Kowal et al. 2016; Melo et al. 2015; Nakamura et al. 2022), effective markers for predicting organ-specific metastasis remain lacking (Li et al. 2024; Zhang et al. 2024). Using our workflow, plasma EV-associated TRAIL showed strong performance in distinguishing PDAC patients with and without liver metastasis and in predicting early postoperative recurrence, which may have important clinical implications for PDAC management.

Despite these strengths, there are several limitations. Ultracentrifugation, although widely used for EV enrichment, may co-isolate lipoproteins and protein aggregates with sizes and densities similar to EVs, potentially affecting background signals and absolute particle counts (Yuana et al. 2014; Sódar et al. 2016; Karimi et al. 2018). Incorporation of orthogonal purification strategies in future studies, such as size-exclusion chromatography or immunoaffinity-based methods, may further improve analytical specificity and reproducibility. In addition, although our phenotyping employed a two-color configuration with minimal spectral overlap, fluorescence compensation remains an important consideration in nanoscale flow cytometry, where low signal amplitudes can increase susceptibility to false-positive calls; rigorous evaluation and application of compensation using appropriate single-stained controls will further strengthen assay robustness in future implementations.

In summary, we present a CytoFLEX-based nanoscale flow cytometry workflow with clearly defined quality-control steps for plasma EV analysis, and we show that EV-associated TRAIL is associated with liver metastasis in PDAC and has value for postoperative risk assessment. By integrating methodological rigor with biological and clinical validation, our approach advances EV-based liquid biopsy strategies toward analytically robust and translationally relevant implementation.

## Supplementary Information

Supplementary Material 1.

Supplementary Material 2.

## Data Availability

These resources are available upon reasonable request to the corresponding authors.
