# Supplementary material for: Detection of plasma EV-associated TRAIL by nanoscale flow cytometry for liver metastasis prediction in PDAC
Source: Adv Biotechnol (Singap). 2026 Mar 4;4(1):6. doi: 10.1007/s44307-026-00102-1 (PMC12961005; doi:10.1007/s44307-026-00102-1)

**Supplementary Materials**

**Detection of Plasma EV-associated TRAIL by Nanoscale Flow Cytometry for Liver Metastasis Prediction in PDAC**

Chun-Xiang Huang^1,2^, Jia-Hong Jian^1,2^, Jun-Sheng Hao ^1,2^, Zi-Wen Zhou^1^, Zhuo-Qun Li^1^, Dong-Ming Kuang^1,*^, Cai-Yuan Wu^1,3,*^

^1^Guangdong Province Key Laboratory of Pharmaceutical Functional Genes, MOE Key Laboratory of Gene Function and Regulation, School of Life Sciences, Sun Yat-sen University, Guangzhou 510275, China

^2^These authors contributed equally

^3^Lead contact

*Correspondence: kdming@mail.sysu.edu.cn (D.-M.K.), wucy23@mail.sysu.edu.cn (C.-Y.W.)

This PDF file includes:

Supplementary Figures (Supplementary Fig.1)

Source data fpr Immunoblotting

**Supplementary Fig.1:**


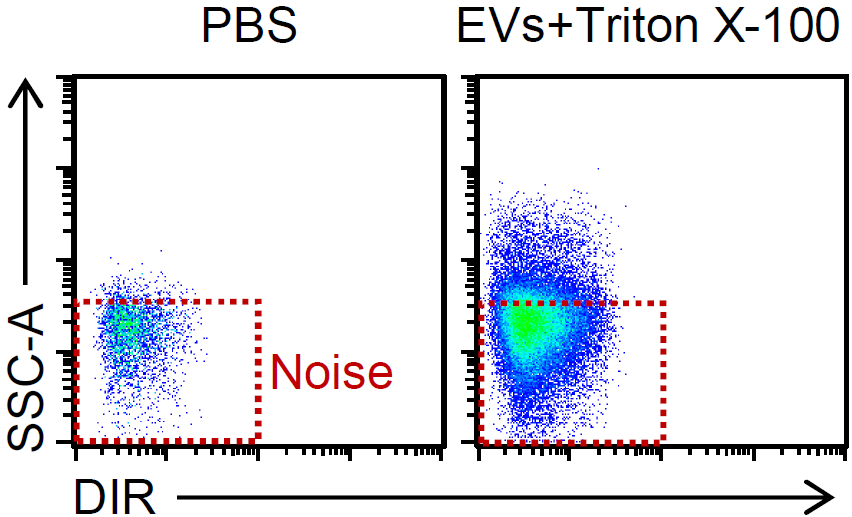


Representative CytoFLEX plots of particle-free PBS and DiR-labeled HEK293T EVs after Triton X-100 treatment (detergent lysis control). Experiments were repeated independently three times with similar results (n = 3).

**Full uncropped Gels and Blots images:**


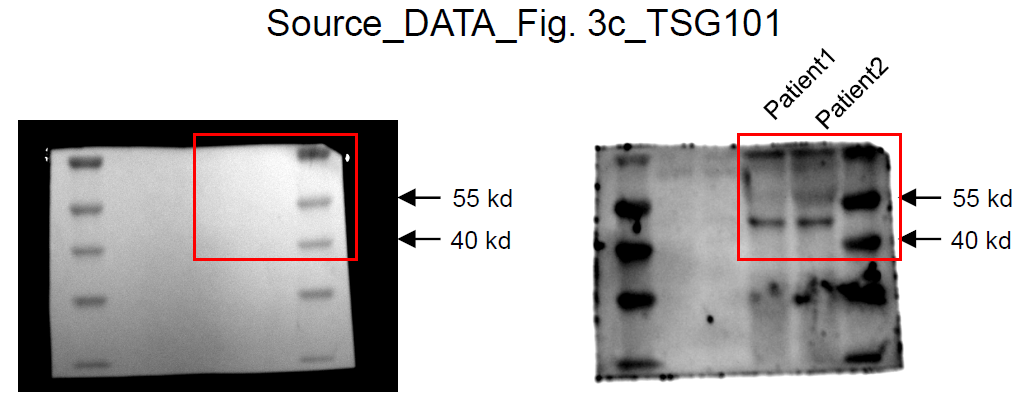


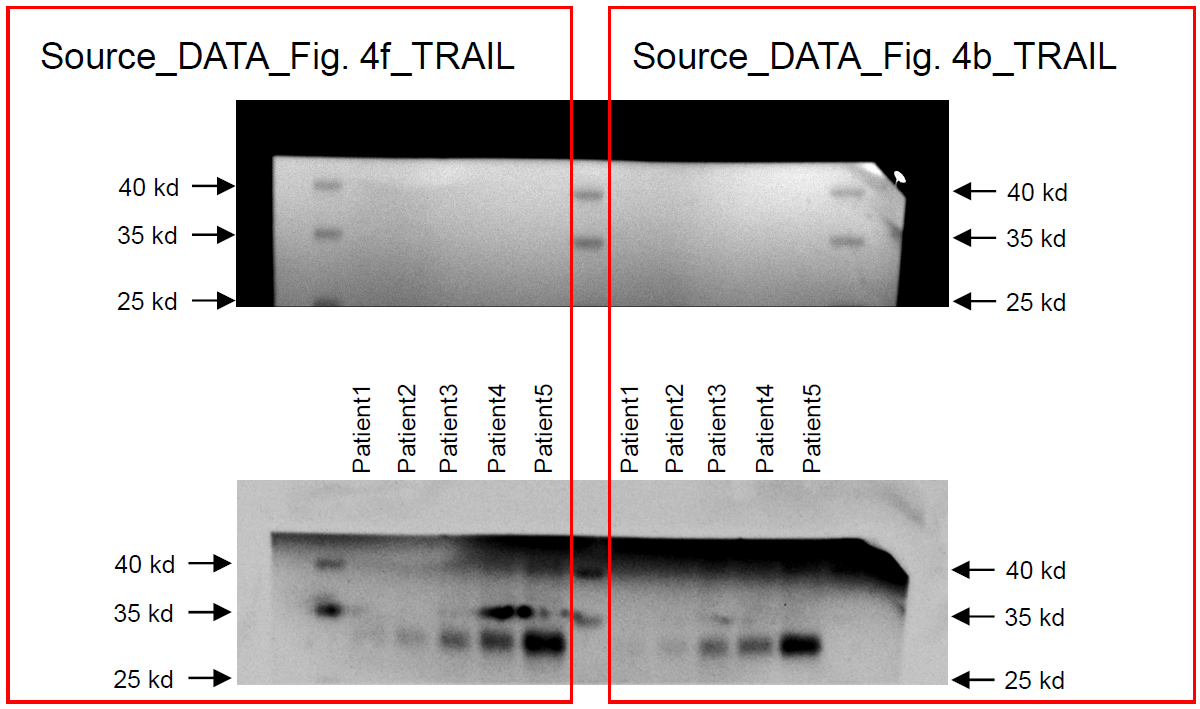

Supplement: Supplementary file 1 — Supplementary Material 1. [file 44307_2026_102_MOESM1_ESM.docx]
